# Blood Transfusion and ABO Blood Groups in Sickle Cell Disease

**DOI:** 10.3390/medicina62081449

**Published:** 2026-07-26

**Authors:** Mortadah Alsalman, Muthana AlSahlawi, Naushad Abid, Khaled Elzorkany, Eman Abdallah, Alaa AlQuraini, Hussain Abduljaleel Alkhalifa, Marwa Shafey, Zaenb Alsalman

**Affiliations:** 1 Department of Medicine, College of Medicine, King Faisal University, Al-Ahsa 31982, Saudi Arabia; moalsalman@kfu.edu.sa (M.A.); mualsahlawi@kfu.edu.sa (M.A.); nabid@kfu.edu.sa (N.A.); kelzorkany@kfu.edu.sa (K.E.); eaali@kfu.edu.sa (E.A.); 2 College of Medicine, King Faisal University, Al-Ahsa 31982, Saudi Arabia; 224009557@student.kfu.edu.sa; 3 Department of Medicine, King Fahad Specialist Hospital, Dammam 32253, Saudi Arabia; hussain-alkhalifa@outlook.com; 4 Department of Family and Community Medicine, College of Medicine, Imam Abdulrahman bin Faisal University, Dammam 31441, Saudi Arabia; mmashafey@iau.edu.sa; 5 Department of Family and Community Medicine, College of Medicine, King Faisal University, Al-Ahsa 31982, Saudi Arabia

**Keywords:** sickle cell disease, blood transfusion, ABO blood group, Rh blood group, hydroxyurea

## Abstract

*Background and Objectives*: Blood transfusion is a key component of sickle cell disease (SCD) management, and ABO and Rh compatibility is essential to minimize transfusion-related complications. This study investigated factors associated with blood transfusion history and examined the relationships of ABO and Rh blood groups with clinical and laboratory markers of disease severity. *Materials and Methods*: A cross-sectional study was conducted among 309 patients attending a hematology outpatient clinic in Saudi Arabia between October and December 2025. Clinical data were collected through patient interviews, and laboratory results were obtained from medical records. *Results*: Overall, 89.9% of patients had a history of blood transfusion. Older age, chronic disease, higher platelet count, MCV, MCH, and HbA2 levels were associated with blood transfusion history. In contrast, higher hemoglobin and HbF levels were associated with fewer transfusions. Patients with blood group O had lower odds of transfusion than those with non-O blood groups (OR = 0.340, 95% CI: 0.135–0.856; *p* = 0.022). Hydroxyurea use was associated with lower LDH levels, while G6PD deficiency and higher MCV were independently associated with higher LDH levels. ABO and Rh blood groups were not associated with acute chest syndrome or LDH levels. *Conclusions*: Blood transfusion history in SCD was associated with several clinical and hematological factors. These findings support further investigation of G6PD status and reinforce the known benefits of hydroxyurea. Further studies are needed to clarify the role of ABO and Rh blood groups in disease progression and transfusion practices.

## 1. Introduction

Sickle Cell Disease (SCD) is an inherited hematological disorder that represents an increasing global health burden, affecting approximately 300,000 births annually, with higher prevalence in sub-Saharan Africa, South Asia, the Middle East, and the Mediterranean regions [1,2]. In Saudi Arabia, the disease is particularly common, affecting around 20,000 individuals per 100,000 population, with the highest rates reported in the Eastern and Southern Provinces [3,4]. It results from a mutation in the beta-globin subunit of hemoglobin, leading to the production of abnormal hemoglobin S (HbS), which tends to polymerize under hypoxic conditions [2,5].

Clinically, SCD manifests with a wide range of complications, including stroke, acute chest syndrome (ACS), splenic sequestration crises, and multi-organ dysfunction [6]. The two core features of the disease are chronic hemolytic anemia and recurrent vaso-occlusive crises (VOC), while ACS remains the leading cause of mortality among affected patients [7,8]. The clinical presentation of SCD is highly diverse and heterogeneous, making the assessment and prediction of disease severity particularly challenging [9].

Previous studies have identified several genetic, hematological, and environmental factors that influence disease severity. Genetically, patients with HbSS and HbS/β^0^ thalassemia genotypes generally experience more severe manifestations than those with other genotypes, while co-inheritance of alpha thalassemia and higher fetal hemoglobin levels can significantly modify disease severity [5,10].

Additionally, the influence of the ABO blood group system on the clinical course of SCD has gained increasing interest because of its role in vascular and coagulation processes [11,12,13,14]. Individuals with non-O blood groups typically exhibit higher circulating levels of von Willebrand factor (vWF) and factor VIII than those with blood group O, which may promote a prothrombotic state and influence vascular function [11,12,14]. These biological mechanisms provide a plausible basis for investigating the potential association between ABO blood groups and disease severity in patients with SCD [13].

Furthermore, G6PD deficiency frequently coexists with SCD in regions where both disorders are highly prevalent [15,16]. While SCD is characterized by chronic oxidative stress and hemolysis, the added impact of G6PD deficiency impairs the erythrocyte’s ability to manage oxidative damage, which may contribute to increased hemolysis and potentially more severe clinical manifestations [15,16,17]. Despite this biological rationale, clinical findings on the impact of G6PD deficiency in SCD remain inconsistent [18,19].

In addition, hematological parameters such as hemoglobin level, white blood cell count (WBC), and platelet count have been strongly associated with SCD-related complications, including ACS and VOC, both of which are considered important indicators of more severe disease progression [9,20]. Socioeconomic factors, particularly access to healthcare, may also influence disease severity and clinical outcomes in patients with SCD [21].

A variety of therapeutic approaches are available for the management of SCD, aiming to improve quality of life, reduce complications, and potentially enhance survival [6,22]. Nevertheless, red blood cell transfusion, whether administered as simple or exchange transfusion, remains a cornerstone in the management of SCD and plays a critical role in preventing and treating severe disease-related complications [23,24]. However, the factors associated with increased blood transfusion in patients with SCD remain incompletely understood. Therefore, this study aimed to identify predictors of blood transfusion history among patients with SCD and to evaluate the associations of ABO and Rh blood groups with blood transfusion history and clinical and laboratory markers of disease severity.

## 2. Materials and Methods

### 2.1. Study Population

A cross-sectional study was conducted among patients at a hematology outpatient clinic in Saudi Arabia between October and December 2025. Eligible participants were adults aged 18 years or older with a confirmed diagnosis of SCD who provided informed consent. Patients with incomplete medical records that prevented assessment of key study variables and those who declined participation were excluded. Consecutive sampling was used, and all eligible patients attending the center during the study period were invited to participate until the target sample size was reached.

### 2.2. Data Collection

Data were collected by trained research team members through direct patient interviews using a structured questionnaire. The questionnaire consisted of three main sections. The first section included sociodemographic information such as age, sex, and history of chronic diseases. The second section addressed clinical characteristics of SCD, including annual frequency of VOC, history of ACS, self-reported lifetime number of transfusion episodes, hydroxyurea use, and previous surgical interventions. The third section included laboratory parameters obtained from patients’ medical records, including WBC, hemoglobin level, platelet count, mean corpuscular volume (MCV), mean corpuscular hemoglobin (MCH), HbA, HbA2, HbF, HbS, ABO blood group, Rh D status and lactate dehydrogenase (LDH) levels.

### 2.3. Outcome Definitions

The study outcomes included clinical complications and markers of disease severity in patients with SCD. These comprised a history of blood transfusion (yes/no), self-reported lifetime number of transfusion episodes, a history of ACS, annual frequency of VOC, and LDH levels.

A history of blood transfusion was defined as any self-reported lifetime history of receiving a blood transfusion, regardless of whether it was administered for an acute indication or as part of chronic transfusion therapy. The lifetime number of blood transfusion episodes was also self-reported. A history of ACS was based on participants’ self-report (yes/no). Annual VOC frequency was defined as the self-reported number of VOC episodes experienced during the preceding 12 months. LDH values were obtained from routine laboratory investigations recorded in the patients’ electronic medical records during outpatient follow-up visits and represented the most recent outpatient measurement available during the study period.

### 2.4. Statistical Analysis

Data were entered and analyzed using IBM SPSS Statistics for Windows, version 28.0 (IBM Corp., Armonk, NY, USA). Continuous variables were summarized using mean ± standard deviation or median (IQR), as appropriate. Categorical variables were presented as frequencies and percentages.

Normality was assessed using the Shapiro–Wilk test. Associations between study variables and the study outcomes (blood transfusion history, ACS, annual frequency of VOC, and LDH level) were assessed using the chi-square test (or Fisher’s exact test) for categorical variables and the independent *t*-test or Mann–Whitney U test for continuous variables, as appropriate. Pearson correlation analysis was performed to examine the association between LDH levels and continuous predictors.

Univariate logistic regression analyses were performed to identify factors associated with blood transfusion history and acute chest syndrome (ACS). A multivariable logistic regression model was performed for ACS only, as a multivariable model was not performed for blood transfusion history because of the highly imbalanced binary outcome. Univariate and multivariable linear regression analyses were conducted to identify predictors of LDH levels. Variables included in the multivariable models were selected based on clinical relevance, directed acyclic graph (DAG) considerations, a *p*-value < 0.20 in univariate analyses, and the absence of significant multicollinearity. Statistical significance was set at *p* < 0.05.

## 3. Results

### 3.1. Demographic and SCD Characteristics

A total of 309 patients with SCD were included in the study. The mean age was 33.4 ± 11.7 years, with a median of 32 years (IQR: 25–40; range: 17–88), and more than half were female (55.3%). Nearly one-quarter (24.9%) had at least one chronic comorbidity, while G6PD deficiency was present in 6.5% of patients. A history of stroke was reported in 7.4% of the study, and 31.1% had undergone previous surgical procedures. At the time of assessment, 42.1% were receiving hydroxyurea therapy. A history of VOC was reported in 94.5% of patients, with a median of 3 episodes (IQR: 2–6) and a mean of 5.4 ± 6.5 (range: 1–50). Regarding blood group distribution, blood group O was more common than non-O blood groups (60.8% vs. 39.2%), and 94.2% of patients were Rh D positive. Among the 309 SCD patients, 278 (89.9%) reported a history of blood transfusion. The median number of lifetime transfusion episodes was 5 (IQR: 2–11), with a mean of 9.2 ± 11.2 (range: 1–60). Slightly more than half, 159 (51.5%), had a history of ACS. The mean LDH level was 429.0 ± 231.3 U/L (51.0–2148), while the median was 372 U/L (IQR: 277-496).

Patients with a history of blood transfusion were significantly older than those without a history of transfusion. Patients with chronic comorbidities were significantly more likely to report a history of blood transfusion, and those with non-O blood groups also reported a history of blood transfusion (*p* < 0.05). These patients also had a higher annual frequency of VOC (*p* < 0.001). A history of ACS was significantly associated with G6PD deficiency, previous history of surgery, current hydroxyurea use, and a higher annual frequency of VOC. Mean LDH levels were significantly higher in patients with non-O blood groups compared with those with blood group O (*p* < 0.05) (Table 1).

### 3.2. Hematological and Biochemical Parameters

Hematological and biochemical parameters are presented in Table 2. The mean hemoglobin level was 9.36 ± 1.67 g/dL. Hemoglobin electrophoresis showed predominance of HbS, with mean HbF and HbA2 levels of 14.5 ± 7.4% and 3.1 ± 1.1%, respectively. A history of blood transfusion was significantly associated with lower hemoglobin levels, higher platelet counts, increased MCV and MCH values, and lower HbF levels (*p* < 0.05). Meanwhile, higher hemoglobin levels, higher platelet counts, higher HbA, HbA2 and HbS were significantly associated with a history of ACS among SCD patients (*p* < 0.05). Regarding LDH, significant positive correlations were observed with WBC count (r = 0.209, *p* < 0.001), MCV (r = 0.264, *p* < 0.001), and MCH (r = 0.237, *p* < 0.001). In contrast, LDH levels showed a significant negative correlation with HbA2 (r = −0.128, *p* = 0.024) (Table 2).

The number of blood transfusion episodes showed significant positive correlations with WBC count (r = 0.171, *p* = 0.004), platelet count (r = 0.118, *p* = 0.050), and hemoglobin level (r = 0.185, *p* = 0.002). Conversely, significant negative correlations were observed with HbA (r = −0.067, *p* = 0.005) and HbF (r = −0.371, *p* < 0.001). In addition, the presence of Rh D antigen was significantly associated with the average number of blood transfusion episodes (Mann–Whitney Z = −2.140, *p* = 0.032). No statistically significant differences were observed between the studied variables and the remaining outcomes, including the annual number of VOC.

### 3.3. Factors Associated with Blood Transfusion, ACS and LDH Levels

Table 3 summarizes the results of the univariate logistic and linear regression analyses examining factors associated with blood transfusion history, ACS and LDH levels.

Older age was associated with higher odds of blood transfusion history (OR = 1.119, *p* < 0.001; 95% CI: 1.059–1.184). In addition, patients with chronic diseases were more likely to have a history of transfusion (OR = 3.386, *p* = 0.049; 95% CI: 1.000–11.468). ABO blood group was also associated with transfusion history, as patients with blood group O had lower odds of transfusion compared with non-O blood groups (OR = 0.340, 95% CI: 0.135–0.856; *p* = 0.022). Lower hemoglobin levels were associated with a greater likelihood of transfusion (OR = 0.716, 95% CI: 0.565–0.907; *p* = 0.006), while higher platelet counts showed a positive association (OR = 1.003, 95% CI: 1.000–1.005; *p* = 0.039). Higher MCV and MCH values were also associated with increased odds of transfusion history. HbA2 was associated with higher odds of transfusion (OR = 1.587, *p* = 0.040), whereas higher HbF levels were associated with lower odds of blood transfusion (OR = 0.941, *p* = 0.016).

For ACS, G6PD deficiency, higher frequency of VOC, history of surgery, and current use of hydroxyurea were associated with higher odds of ACS. Higher hemoglobin levels and platelet counts were also associated with increased odds of ACS. In contrast, higher levels of HbA were associated with lower odds of ACS (OR = 0.986, *p* = 0.032), whereas higher HbA2 and HbS levels were significantly associated with higher odds of ACS (OR = 1.358, *p* = 0.007 and OR = 1.017, *p* = 0.022, respectively). Neither ABO blood group nor Rh D antigen was significantly associated with ACS in the univariate analysis (*p* > 0.05). Patients with the O blood group had similar odds of ACS compared to those with non-O blood groups (OR = 0.618, 95% CI: 0.516–1.290, *p* = 0.384). Likewise, the presence of Rh D antigen was not significantly associated with ACS (OR = 2.217, 95% CI: 0.810–6.068, *p* = 0.121). For LDH levels, current hydroxyurea use was associated with lower LDH levels (B = −54.869, *p* = 0.039). In contrast, higher WBC count (B = 6.381, *p* = 0.007; 95% CI: 1.770–10.991), MCV (B = 3.451, *p* = 0.001; 95% CI: 1.355–5.546), and MCH (B = 9.123, *p* < 0.001; 95% CI: 3.727–14.520) were significantly associated with higher LDH levels. Meanwhile, HbA2 was associated with lower LDH levels (B = −32.125, *p* = 0.009; 95% CI: −56.096 to −8.153).

In the adjusted multivariable analysis, G6PD deficiency was the strongest independent predictor of ACS (AOR = 4.56, 95% CI: 1.09–19.13, *p* = 0.038), followed by prior surgery (AOR = 3.22, 95% CI: 1.72–6.00, *p* < 0.001), hydroxyurea use (AOR = 2.23, 95% CI: 1.26–3.94, *p* = 0.006), higher frequency of VOC (AOR = 1.17, 95% CI: 1.09–1.26, *p* < 0.001), and higher platelet count (AOR = 1.002, 95% CI: 1.000–1.003, *p* = 0.031). In the adjusted multivariable linear regression analysis, chronic disease and hydroxyurea use remained significantly associated with lower LDH levels (B = −73.82, *p* = 0.033; B = −55.93, *p* = 0.044, respectively). Conversely, G6PD deficiency and higher MCV were independently associated with higher LDH levels (B = 135.85, *p* = 0.025 and B = 2.80, *p* = 0.021, respectively).

## 4. Discussion

Red blood cell transfusion remains a cornerstone in the management of sickle cell disease (SCD), particularly for the prevention and treatment of acute complications. However, the cumulative burden of iron overload, alloimmunization, and infection risk remains a substantial challenge for both clinicians and patients [23,25]. Despite international guidelines, transfusion practices vary widely by region [26,27]. Our study highlights the high prevalence of transfusion history and reinforces the concept that transfusion needs are multifactorial rather than driven by a single clinical marker.

SCD is a multisystem disorder with a remarkably diverse clinical spectrum. Currently, there is no universally accepted tool for the objective and reproducible assessment of disease severity, a gap that complicates efforts to optimize treatment outcomes [22,28]. Notably, although the mean age of participants in our study was thirty, the lifetime transfusion rate was lower than the annual rates reported in the previous literature [29]. Furthermore, our findings suggest that transfusion history increases with age. The observed association likely reflects cumulative disease exposure, rather than age being an independent predictor. Younger patients inherently have had fewer years living with SCD and, therefore, fewer opportunities to receive transfusions.

Historically, elevated fetal hemoglobin (HbF) levels and the coexistence of alpha thalassemia, often reflected by lower mean corpuscular volume (MCV) and mean corpuscular hemoglobin (MCH), have been recognized as important modifiers of SCD severity [30]. In our study, higher HbF levels were associated with lower blood transfusion history despite lower LDH levels, consistent with the protective role of HbF reported in previous studies [31,32]. Conversely, coexisting alpha thalassemia is a well-established modifier of SCD progression. Our findings showed that higher MCV and MCH values were associated with a history of blood transfusion and higher hemolytic activity; notably, these indices are typically lower in patients with alpha thalassemia. Furthermore, we observed that higher HbA2 levels were associated with an increased history of blood transfusion and a higher incidence of ACS. Interestingly, HbA2 also showed a significant negative correlation with LDH levels, which may point toward co-inherited beta-thalassemia trait within our patients. While beta-thalassemia is linked to increased transfusion needs, it often displays an inverse relationship with hemolytic markers. This suggests the involvement of alternative mechanisms of anemia, such as splenic sequestration or hypersplenism secondary to chronic splenomegaly [33,34]. It is also possible that elevated MCV related to reticulocytosis may mask the underlying microcytosis usually associated with alpha or beta thalassemia [35].

Glucose-6-phosphate dehydrogenase (G6PD) deficiency is an X-linked recessive disorder that causes hereditary hemolytic anemia, typically triggered by oxidative stress, although chronic hemolysis may also occur [16,36]. Both G6PD deficiency and sickle hemoglobin are thought to confer partial protection against malaria, which contributes to their high prevalence in malaria-endemic regions. Previous studies have shown that G6PD deficiency may increase transfusion requirements during early childhood in patients with SCD, although findings in older patients remain inconsistent [18,19]. Our results indicate that the coexistence of G6PD deficiency and SCD was associated with increased hemolysis, supporting earlier reports suggesting more severe anemia in patients with both conditions [19,36]. Although donor G6PD status was not evaluated in our study, emerging evidence suggests that G6PD-deficient donor red blood cells may affect transfusion outcomes because of reduced post-transfusion survival and increased susceptibility to oxidative damage [37].

SCD is also characterized by chronic inflammation involving leukocytes, platelets, and endothelial activation, along with impaired resolution of inflammation. In our study, leukocytosis and thrombocytosis were associated not only with increased hemolysis but also with more blood transfusions. Conversely, patients with higher baseline hemoglobin levels tended to require fewer transfusions [6,9]. Furthermore, beyond its established role in preventing acute and long-term complications of SCD, hydroxyurea appeared to reduce disease severity by decreasing hemolytic activity. This finding is consistent with the well-established benefits of hydroxyurea in reducing disease severity and transfusion burden [38,39]. However, the association between hydroxyurea use and ACS should be interpreted cautiously, as it may reflect confounding by indication. Although VOC frequency is an important marker of SCD severity, it was significantly associated with ACS but not with blood transfusion history in our study. This may reflect the multifactorial nature of transfusion decisions, which depend on several clinical and laboratory factors.

Regarding ABO and Rh blood group systems, we found that blood group O was the most common type among patients with SCD, which aligns with previous epidemiological reports. This finding is expected, as blood group O and Rh D positive are the predominant blood groups in the Eastern Province of Saudi Arabia [40,41]. While blood group variations have increasingly been associated with non-transfusion-related conditions, such as nephropathy and venous thromboembolism, our data suggest that patients with non-O blood groups were more likely to have a history of blood transfusion than those with blood group O [42,43]. This supports the hypothesis that non-O individuals, who typically exhibit higher circulating levels of von Willebrand factor and factor VIII, may experience a more complex clinical course [41]. In addition, Rh D positive individuals had higher transfusion rates than Rh D negative individuals; however, this finding should be interpreted cautiously because the relatively small number of Rh D negative participants may have limited the statistical power of the analysis. Importantly, there was no observable influence of blood group on the incidence of acute chest syndrome and acute painful crisis.

This study underscores the important role of blood transfusion in the management of SCD, with most patients requiring at least one transfusion during their lifetime. Our findings suggest that factors such as higher HbF levels and hydroxyurea use may contribute to a lower transfusion burden, which could partly explain the lower transfusion rates observed in our study compared with previous studies. However, this study is not without limitations. The cross-sectional design limits conclusions regarding causality, and the retrospective collection of clinical information may have introduced recall and documentation bias. The retrospective design also prevented us from determining whether ACS occurred before or after hydroxyurea treatment was initiated. Additionally, the high baseline rate of blood transfusions resulted in a highly skewed outcome distribution, which statistically limited our ability to perform fully adjusted multivariable logistic regression models without risking model instability, overfitting, or coefficient inflation. Future studies may consider penalized logistic regression and assess transfusion burden using objectively documented transfusion records. Furthermore, genetic testing and measurements of von Willebrand factor (vWF) and factor VIII were not routinely available, limiting our ability to assess their potential contribution to disease severity and transfusion requirements. Future research should examine these markers, include both patient and donor G6PD status, compare adherence to transfusion guidelines, evaluate restrictive versus liberal transfusion strategies across different regions, include appropriate control populations for ABO and Rh D comparisons, and further clarify the role of ABO and Rh blood groups in SCD progression and transfusion practices.

## 5. Conclusions

Blood transfusion history in patients with SCD was associated with several clinical and hematological factors, including age, chronic comorbidities, hemoglobin level, platelet count, and HbF. Hydroxyurea use was independently associated with lower LDH levels, while G6PD deficiency was associated with higher LDH levels, highlighting their relationship with hemolysis. These findings reinforce the importance of adherence to hydroxyurea therapy and suggest a potential role for G6PD screening in the comprehensive management of SCD. Patients with non-O blood groups were also more likely to have a history of blood transfusion than those with blood group O. However, no significant association was observed between ABO or RhD blood groups and ACS. Further prospective studies are needed to clarify the role of ABO and Rh blood groups in SCD progression and transfusion practices.

## Figures and Tables

**Table 1:** Differences in blood transfusion history, ACS and LDH levels according to Demographic and Clinical Characteristics (*n* = 309).

Characteristic	Total *n* (%)	History of Blood Transfusion		History of Acute Chest Syndrome		LDH^ £^
		No 31 (9.1%) Yes 278 (89.9%)		No 150 (48.5%) Yes 159 (51.5%)		(Mean = 429.01)
		*p*-Value		*p*-Value		*p*-Value
**Age in Years**	33.4 ± 11.7	25.32 ± 7.56	34.31 ± 11.77	33.13 ± 12.17	33.67 ± 11.31	R = 0.096
**(Mean ± SD)**		<0.001 *		0.398		0.091
**Gender**						
Male	138 (44.7)	17 (12.3)	121 (87.7)	70 (50.7)	68 (49.3)	449.1 ± 230.2 (389)
Female	171 (55.3)	14 (8.2)	157 (91.8)	80 (46.8)	91 (53.2)	412.8 ± 231.6 (355)
		0.229		0.491		0.121
**Chronic Diseases**						
No	232 (75.1)	28 (12.1)	204 (87.9)	114 (49.1)	118 (50.9)	432.8 ± 242.9 (372)
Yes	77 (24.9)	3 (3.9)	74 (96.1)	36 (46.8)	41 (53.2)	417.6 ± 193.5 (374)
		0.039 *		0.717		0.877
**G6PD**						
No	289 (93.5)	30 (10.4)	259 (89.6)	146 (50.5)	143 (49.5)	423.3 ± 230.3 (372.0)
Yes	20 (6.5)	1 (5.0)	15 (95.0)	4 (20.0)	16 (80.0)	512.1 ± 236.9 (441.5)
		0.705 ^&^		0.008 *		0.059
**Previous History of Surgery**						
No	213 (68.9)	20 (9.4)	193 (90.6)	126 (59.2)	87 (40.8)	440.3 ± 245.8 (381)
Yes	96 (31.1)	11 (11.5)	85 (88.5)	24 (25.0)	72 (75.0)	403.9 ± 194.1 (360)
		0.575		<0.001 *		0.213
**Current Use of Hydroxyurea**						
No	179 (57.9)	18 (10.1)	161 (89.9)	103 (57.5)	76 (42.5)	252.1 ± 261.0 (386)
Yes	130 (42.1)	13 (10.0)	117 (90.0)	47 (36.2)	83 (63.8)	397.2 ± 178.9 (359)
		0.987		<0.001 *		0.104
History of vaso-occlusive crises (VOC)						
No	17 (5.5)	3 (17.6)	14 (82.4)	9 (52.9)	8 (47.1)	437.3 ± 261.4 (339)
Yes	292 (94.5)	28 (9.6)	264 (90.4)	141 (48.3)	151 (51.7)	428.5 ± 229.9 (373)
		0.282		0.709		0.694
Number of VOC	5.4 ± 6.5	4.8 ± 4.5	5.1 ± 6.6	3.3 ± 3.2	6.8 ± 8.0	R = 0.054
**(Mean ± SD)**		<0.001 *		<0.001 *		0.344
**Blood Parameters and ABO Grouping**						
**ABO grouping**						
Non-O	121(39.2)	6 (5.0)	115 (95.0)	55 (45.5)	66 (54.5)	453.8 ± 219.3 (395)
O	188 (60.8)	25 (13.3)	163 (86.7)	95 (50.5)	93 (49.5)	413.1 ± 238.1 (359)
		0.017 *		0.383		0.040 *
**Rh D Status**						
Rh D neg	18 (5.8)	2 (11.1)	16 (88.9)	12 (66.7)	6 (33.3)	380.9 ± 178.2 (332.5)
Rh D pos	291(94.2)	29 (10.0)	262 (90.0)	138 (47.4)	153 (52.6)	431.9 ± 234.1 (374.0)
		0.875		0.113		0.291

Abbreviations: SCD, sickle cell disease; LDH, lactate dehydrogenase; G6PD, glucose-6-phosphate dehydrogenase. & Fisher’s Exact Test used. £ Mann–Whitney test used. * Significant at *p* < 0.05.

**Table 2:** Differences in blood transfusion history, ACS and LDH levels according to Hematological Parameters and Hemoglobin Fractions Among SCD Patients (*n* = 309).

Blood Parameters	Mean ± SD	Median (IQR 25th–75th)	History of Blood Transfusion No 31 (9.1%) Yes 278 (89.9%) *p*-Value	History of Acute Chest Syndrome No 150 (48.5%) Yes 159 (51.5%) *p*-Value	LDH (Mean = 429.01) *p*-Value
**WBC (×10^9^/L)**	9.33 ± 5.59	8.43 (5.5–11.7)	8.44 ± 3.38 9.44 ± 5.76 0.714	9.21 ± 6.09 9.46 ± 5.1089 0.489	R = 0.209 <0.001 *
**Haemoglobin (g/dL)**	9.36 ± 1.67	9.40 (8.25–10.50)	10.16 ± 1.78 9.27 ± 1.64 0.005 *	9.12 ± 1.68 9.59 ± 1.64 0.014 *	R = −0.072 0.207
**Platelet(×10^9^/L)**	316.21 ± 186.58	272 (165.0–420.50)	249.84 ± 135.29 323.61 ± 190.21 0.036 *	287.04 ± 172.02 343.73 ± 195.93 0.011 *	R = 0.021 0.711
**MCV(fL)**	79.46 ± 12.19	78.60 (70.20–87.45)	75.41 ± 9.74 79.91 ± 12.37 0.05 *	79.16 ± 11.86 79.75 ± 12.68 0.670	R = 0.264 <0.001 *
**MCH(pg)**	27.45 ± 4.73	27.30 (23.90–30.0)	25.83 ± 4.34 27.63 ± 4.74 0.025 *	27.21 ± 4.97 27.68 ± 4.50 0.518	R = 0.237 <0.001*
**Hb A (%)**	8.72 ± 18.04	0 (0–6.75)	2.45 ± 5.13 9.42 ± 18.81 0.279	11.04 ± 20.15 6.53 ± 15.53 0.002 *	R = 0.042 0.462
**Hb A2 (%)**	3.10 ± 1.10	2.80 (2.4–3.5)	2.72 ± 0.61 3.14 ± 1.01 0.124	2.93 ± 1.00 3.26 ± 1.17 0.005 *	R = −0128 0.024 *
**Hb S (%)**	73.62 ± 15.92	77.6 (70.15–82.90)	76.81 ± 6.66 73.27 ± 16.60 0.934	71.46 ± 17.32 75.67 ± 14.21 0.022 *	R = −0.049 0.389
**Hb F (%)**	14.49 ± 7.43	14.60 (8.55–19.50)	17.59 ± 5.67 14.15 ± 7.53 0.014 *	14.5 ± 57.82 14.43 ± 7.07 0.891	R = 0.001 0.990

Mann–Whitney test is used for all; *t* test; Pearson correlation for Haemoglobin, MCV, Hb F. * Significant at *p* < 0.05.

**Table 3:** Univariate logistic and linear regression analysis of factors associated with Blood Transfusion, Acute chest syndrome and LDH Level among SCD patients (*n* = 309).

Characteristic	Blood Transfusion OR (*p*-Value)	Blood Transfusion 95% CI	Acute Chest Syndrome OR (*p*-Value)	Acute Chest Syndrome 95% CI	LDH B Coefficient (*p*-Value)	LDH 95% CI
**Age (years)**	1.119 (<0.001 *)	1.059–1.184	1.004 (0.686)	0.985–1.023	1.880 (0.095)	−0.326–4.086
**Gender** **(Ref: Male)**	1.576 (0.232)	0.747–3.322	1.171 (0.491)	0.747–1.835	−36.37 (0.171)	−88.388–15.639
**Chronic Diseases (Ref: No)**	3.386 (0.049 *)	1.000–11.468	1.100 (0.717)	0.657–1.844	38.579 (0.618)	−75.157–44.727
**G6PD (Ref: No)**	2.201 (0.450)	0.284–17.029	4.084 (0.014 *)	1.333–12.512	88.789 (0.097)	−16.161–193.738
**VOC (Ref: No)**	2.020 (0.291)	0.547–7.461	1.205 (0.709)	0.452–3.209	−0.009 (0.880)	−122.522–104.895
**Average frequency of VOC**	1.010 (0.760)	0.948–1.076	1.165 (<0.001 *)	1.093–1.242	−0.007 (0.906)	−4.300–3.814
**Previous History of Surgery** **(Ref: No)**	0.801 (0.567)	0.368–1.745	4.345 (<0.001 *)	2.540–7.431	−36.400 (0.201)	−92.300–19.500
**Current Use of Hydroxyurea** **(Ref: No)**	1.006 (0.987)	0.474–2.135	2.393 (<0.001 *)	1.504–3.809	−54.869 (0.039 *)	−107.047–−2.691
**ABO grouping (Ref: Non-O)**	0.340 (0.022 *)	0.135–0.856	0.618 (0.384)	0.516–1.290	−40.667 (0.132)	−93.613–12.274
**Rh D antigen** **(Ref: Rh D neg)**	1.129 (0.875)	0.247–5.159	2.217 (0.121)	0.810–6.068	51.036 (0.365)	−59.557–161.630
**WBC**	1.041 (0.340)	0.958–1.131	1.008 (0.684)	0.969–1.050	6.381 (0.007 *)	1.770–10.991
**Haemoglobin**	0.716 (0.006 *)	0.565–0.907	1.187 (0.015 *)	1.034–1.362	−13.454 (0.087)	−28.875–1.966
**Platelet**	1.003 (0.039 *)	1.000–1.005	1.002 (0.008 *)	1.000–1.003	−0.061 (0.386)	−0.200–0.078
**MCV**	1.034 (0.050 *)	1.000–1.007	1.004 (0.669)	0.986–1.023	3.451 (0.001 *)	1.355–5.546
**MCH**	1.093 (0.049 *)	1.002–1.192	1.021 (0.388)	0.974–1.071	9.123 (<0.001 *)	3.727–14.520
**Hb A**	1.046 (0.067)	0.997–1.097	0.986 (0.032 *)	0.973–0.999	0.667 (0.362)	−0.771–2.106
**Hb A_2_**	1.587 (0.040 *)	1.021–2.466	1.358 (0.007 *)	1.087–1.698	−32.125 (0.009 *)	−56.096–−8.153
**Hb F**	0.941 (0.016 *)	0.896–0.989	0.998 (0.891)	0.968–1.028	−1.841 (0.300)	−5.330–1.648
**Hb S**	0.983 (0.244)	0.955–1.012	1.017 (0.022 *)	1.002–1.032	−0.352 (0.672)	−1.984–1.280

* Significant at *p* < 0.05.

## Data Availability

The data supporting the findings of this study are included in the manuscript. De-identified data may be available from the corresponding author upon reasonable request, subject to institutional and ethical approval.
